# Post Translational Modulation of β-Amyloid Precursor Protein Trafficking to the Cell Surface Alters Neuronal Iron Homeostasis

**DOI:** 10.1007/s11064-019-02747-y

**Published:** 2019-02-22

**Authors:** Andrew Tsatsanis, Stuart Dickens, Jessica C. F. Kwok, Bruce X. Wong, James A. Duce

**Affiliations:** 1 School of Biomedical Sciences, Faculty of Biological Sciences, University of Leeds, Leeds, West Yorkshire UK; 2 The ALBORADO Drug Discovery Institute, University of Cambridge, Cambridge Biomedical Campus, Hills Road, Cambridge, UK; 3 Melbourne Dementia Research Centre, The Florey Institute of Neuroscience and Mental Health, The University of Melbourne, Parkville, VIC Australia

**Keywords:** β-Amyloid precursor protein (APP), Iron, Ferroportin (FPN), N-glycosylation, Phosphorylation, Trafficking

## Abstract

Cell surface β-Amyloid precursor protein (APP) is known to have a functional role in iron homeostasis through stabilising the iron export protein ferroportin (FPN). Mechanistic evidence of this role has previously only been provided through transcriptional or translational depletion of total APP levels. However, numerous post-translational modifications of APP are reported to regulate the location and trafficking of this protein to the cell surface. Stable overexpressing cell lines were generated that overexpressed APP with disrupted N-glycosylation (APP^N467K^ and APP^N496K^) or ectodomain phosphorylation (APP^S206A^); sites selected for their proximity to the FPN binding site on the E2 domain of APP. We hypothesise that impaired N-glycosylation or phosphorylation of APP disrupts the functional location on the cell surface or binding to FPN to consequentially alter intracellular iron levels through impaired cell surface FPN stability. Outcomes confirm that these post-translational modifications are essential for the correct location of APP on the cell surface and highlight a novel mechanism by which the cell can modulate iron homeostasis. Further interrogation of other post-translational processes to APP is warranted in order to fully understand how each modification plays a role on regulating intracellular iron levels in health and disease.

## Introduction

β-Amyloid precursor protein (APP) is a type 1 transmembrane glycoprotein with a sequence that is highly conserved in the vertebrate phyla, suggesting an important physiological role [1, 2]. In humans it exists as several different length isoforms, with the shortest containing 695 residues being the predominant neuronal isoform [3, 4]. The greatest research attention on this protein has been its association with Alzheimer’s disease (AD). As the name suggests APP is the precursor to amyloid-β (Aβ), the peptide prominently present within senile plaques, and familial mutations in the APP gene elevate the production of Aβ in early onset AD. Functionally it has been implicated in a multitude of roles ranging from signalling to cell adhesion [5]. However, more recently a role in neuronal iron homeostasis has also been proposed, whereby cell surface APP can stabilise the iron export protein ferroportin (FPN), to enable cellular iron efflux through this pore protein [6–10]. All prior evidence of this iron regulatory role in APP has come from reports in which total APP has been ablated by transcriptional knockout or translational knockdown. However, APP is known to have numerous post-translational modifications (PTMs) that regulate location and trafficking throughout the cell. Most heavily researched are the proteolytic processing pathways of APP, largely due to Aβ being a product from the amyloidogenic cleavage of APP. However, before APP reaches the cell surface and is predominantly directed down these processing pathways, a series of other PTMs are required for its transport from the endoplasmic reticulum (ER) to the cell surface. As APP is considered to have a main functional location on the cell surface, trafficking to this location is a key regulator in the function of the full length protein as well as the downstream production of cleavage products such as Aβ.

Broadly, APP is synthesised in the ER and transported to the Golgi, where it enters the trans-Golgi network (TGN) before it is trafficked to and from the cell surface through several cellular compartments. How long APP is held in each compartment can thus determine the proportion of APP that is present on the cell surface and have a regulatory control in the function of APP, such as binding and stabilising FPN in the case of iron efflux. During trafficking of APP a wide range of PTMs extensively alter cellular localisation and their ablation can prevent APP from moving between compartments (for review see [11]). The effects of glycosylation and phosphorylation have been the focus of most investigation in this area, but APP is also known to be palmitoylated, ubiquitinated, sumoylated and sulphated. Several of these modifications may have a close interplay as has been shown with other transmembrane proteins [12, 13]. Unglycosylated APP is referred to as immature as only glycosylated APP is present on the cell surface and subsequently secreted [14, 15]. APP can undergo both N- and O-linked glycosylation. N-glycosylation of APP originates in the ER, from which it is relocated to the Golgi/TGN, where an O-glycosylation is added along with a number of other PTM’s (e.g. sulfation, palmitolyation and phosphorylation) [16]. N-glycosylation occurs at two asparagine residues within the E2 domain of APP; N467 and N496 on APP^695^ [17] and is essential for APP trafficking [15, 18–20]. The fully glycosylated APP has a preference to also be phosphorylated with several sites in both the extracellular and cytoplasmic domains. While more studies about APP phosphorylation have focused on residues within the cytoplasmic domain, two serine residues in the ectodomain are also phosphorylated under basal conditions. Intracellular phosphorylation of serine 196 and 206 on APP^695^ (S196 and S206) are both necessary for APP trafficking [21] but can also be phosphorylated on the cell membrane, indicating a function distinct from trafficking [22].

To explore whether PTMs of APP were able to alter the normal functional role of cell surface APP in iron homeostasis, we focused on modifying N-glycosylation and ectodomain phosphorylation sites located near the previously identified FPN binding sequence in the E2 domain of APP (amino acids 327–348 on APP^695^) [6]. We hypothesised that impaired N-glycosylation and phosphorylation at these sites would either disrupt location of APP on the cell surface or binding of APP to FPN and have a consequential effect of intracellular iron levels through impaired cell surface FPN stability. Neuroblastomas stably overexpressing either wild-type APP^695^ or where the N-glycosylation sites or S206 phosphorylation site were mutated were investigated for cellular location of APP and FPN as well as intracellular iron levels.

## Methods

### Generation and Maintenance of Stable APP Cell-Lines

N2a neuroblastoma cell-lines were maintained in DMEM media (Lonza) containing 10% fetal bovine serum at 37 °C and 5% CO_2_. The phosphorylation (S206A) and N-glycosylation (N467K and N496K) mutations in APP^695^ were generated through PCR-derived mutagenesis of wild-type-APP^695^ in pIRES*hyg* (BD Biosciences) using the QuikChange II XL Site-Directed Mutagenesis Kit (Agilent Technologies). Ablation of the phosphorylation site at S206 was carried out by designing primers that substituted nucleotide T–G at position 616, ablation of the gylcosylation site at N467 was by substituting nucleotide T–G at position 1401 and ablation of the gylcosylation site at N496 was by substituting nucleotide C–G at position 1488. Both protein and DNA sequences are in reference to the 695 isoform sequence. Transiently transfected cells were checked for viability before stable transfected N2a neuroblastoma cell-lines for wild-type-APP^695^ (APP^WT^), the APP mutants (APP^S206A^, APP^N467K^ and APP^496K^) and pIRES*hyg* empty vector were generated by electroporation (250V; 1650 µF). In the presence of hygromycin B (250 µg/mL), 30 µg of plasmid was used to transfect cells within a 75 cm^2^ flask. After 24 h media was replaced with DMEM + 10% FBS and cells were allowed to grow to 80% confluency. At this point selection of successfully transfected cells were maintained by the addition of hygromycin B to the media (DMEM + 10% FBS containing hygromycin B, 250 µg/mL). Clones for each stably incorporated transgene were selected based on comparable expression levels to clones of the other constructs.

### Antibodies

For APP, recognition of N-terminal epitopes was with 22C11 (1:50, Millipore UK Ltd, Livingston, UK) or AB15272 (1:50; Abcam, Cambridge, UK) whereas sAPPβ was detected with 1A9 (1:2500; provided by I. Hussain, Glaxo- SmithKline, Harlow, UK) [23]. Protein response to iron was detected with rabbit anti-Ferritin (1:1000, Cell Signalling Technology). Ferroportin location was identified using rabbit anti-FPN (1:50; BioScience Life Sciences). Loading control was established with mouse anti-β-actin (1:5000, AC15, Sigma). The fluorescently labeled secondary antibodies Alexa Fluor 488 Goat anti-Rabbit IgG and Alexa Fluor 488 Goat anti-Mouse IgG were from Molecular Probes (1:200; Life Technologies).

### Immunoblotting

After each experimental condition media was collected and concentrated using Microcon-30 kDa condensers (Millipore; 30 kDa cut off), while cells were washed twice with cold PBS before collection and homogenised in RIPA buffer (150 mM NaCl, 1% (v/v) Nonidet P-40, 1% (w/v) sodium deoxycholate, 0.1% (v/v) SDS, 25 mM Tris–HCl, pH 7.6) with EDTA-free protease inhibitor cocktail (Complete; Roche). Lysates were clarified by centrifugation at 14,000×*g* for 15 min.

As determined by BCA assay, 10 µg total protein in media or cell lysate from each experimental condition was separated either on 10% PAGE (Tris–Glycine, BioRad) for detection of sAPP/APP (22C11 or 1A9) and 4–20% PAGE (Tris–Glycine, BioRad) for FT. Resolved proteins transferred to polyvinylidene difluoride membranes (Hybond-P, GE Healthcare Life Sciences) were probed with primary and secondary antibodies before visualization with ECL (ThermoScientific) using a LAS-3000 Imaging suite. Densitometry using Image J (NIH) was performed in triplicate on three separate experiments unless otherwise stated and all quantitation was standardized against β-actin levels.

### Fluorescence-Activating Cell Sorting (FACS) Analysis

Neuroblastoma lines were lightly-fixed (1% paraformaldehyde) and assayed by flow cytometry (FACS) for cell-surface bound APP and FPN. Antibodies raised against the extracellular domain epitope for APP (AB15272) or FPN were used. Cells were sorted by forward and side scatter on a BD-LSR-Fortessa (BD Biosciences) according to fluorescence at 530 ± 30 nM. Upon gating to ensure only a live cell population was monitored, a minimum of 10,000 cells were recorded in each experiment, duplicated and repeated in three separate experiments. Data were analyzed using BD FACS DiVa 6 and FlowJo 7.6.4 software.

### Analysis of Labile Iron Pool

The cytoplasmic labile iron pool of all stable cell-lines were measured by a Calcein-AM assay adapted from a previously reported procedure [24]. Briefly, cells were plated at 20,000 cells/well in a black 96-well microplate before treatment with each experimental condition. Cells were washed twice with PBS after which Calcein-AM (60 nM) was added. Fluorescence at an excitation of 485 nm and emission of 535 nm measurements were started immediately and taken every minute using a Biotek Fluorescence microplate reader for 10 min or until a consistent minimum reading had been reached. In triplicate, deferiprone was rapidly added and fluorescence measurements started again immediately. Readings were continued for a further 5 min or until a plateau had been reached. The percentage labile iron pool chelated was calculated as the ΔF (the difference in fluorescence from before and after chelator addition) for each condition compared to the APP^WT^ overexpressing control under endogenous iron homeostatic regulation.

## Results

### APP Mutants Reduce Trafficking to the Cell Surface Reducing APP Degradation

**Fig. 1:**
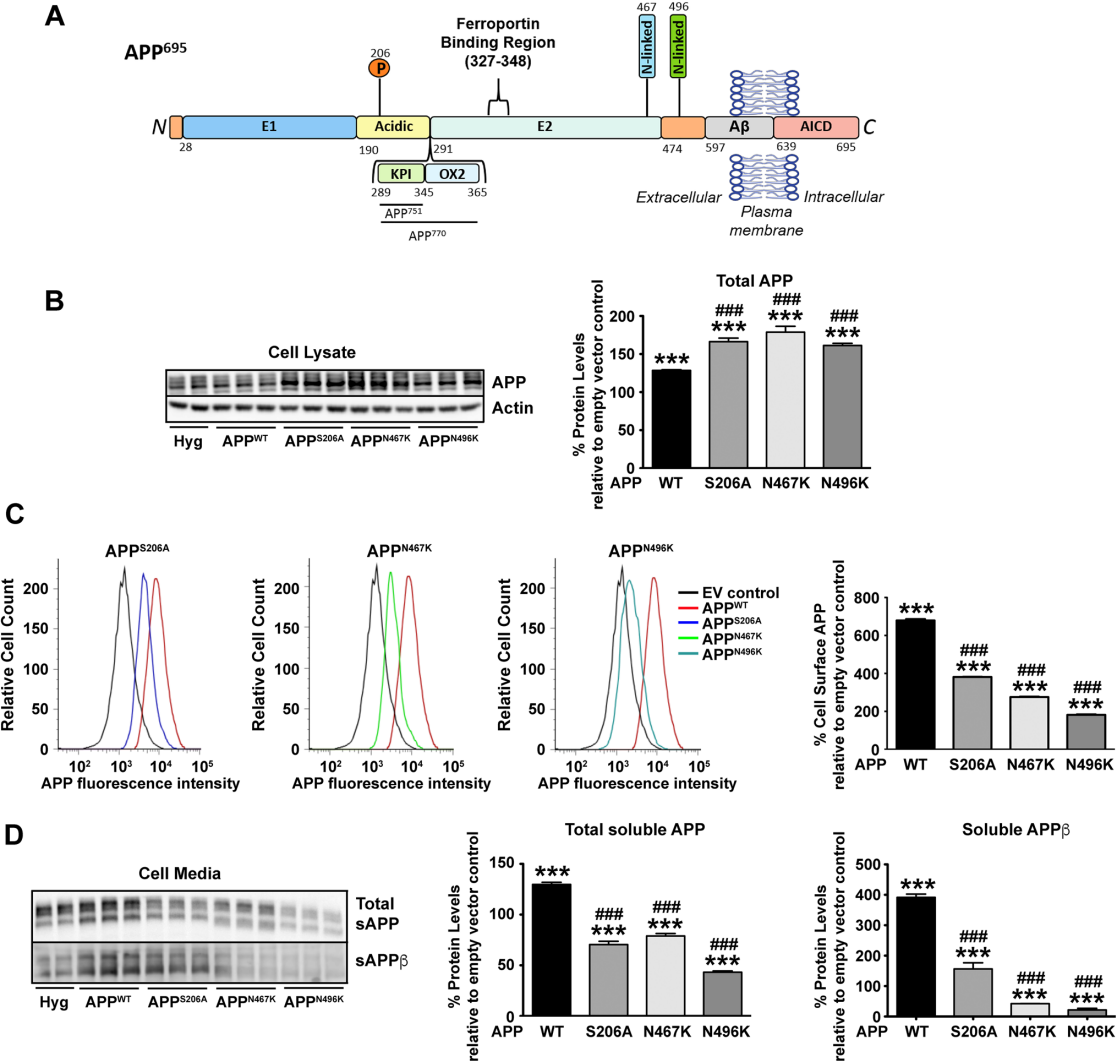
Single point mutations in APP that ablate ectodomain N-glycosylation and phosphorylation sites in APP alter its cellular location and proteolytic processing. N2a cells were stably transfected with wild-type APP (WT) or APP carrying a mutation in a known site of phosphorylation (S206A) or N-glycosylation (N467K and N496K). **a** An amino acid schematic showing the domains and relevant PTM sites of APP^695^. The ferroportin binding region is located inside the E2 domain (amino acids 327–348) [6]. Within the ectodomain the phosphorylation site (206) is located in the acidic domain and the two N-linked glycosylation sites (467 and 496) are located just before and after the end of the E2 domain. Numbering is based on the amino acid sequence of the 695 amino acid isoform that is prevalent in neurons. From the three most common isoforms of APP, the 695 amino acid form lacks the Kunitz protease inhibitor (KPI) and OX-2 antigen domain, whereas a 751 only possesses only the KPI domain and a 770 isoform contains both domains. **b** Changes in total cellular wild-type APP levels were quantified by Western blotting using an antibody that recognised the N-terminal (22C11). **c** Changes in trafficking of APP to the cell surface caused by the insertion of each mutation was evaluated by flow cytometric quantification of APP on the cell surface of non-permeabilised cells. A shift to the left in the representative histograms towards the empty vector (EV) control cells indicates a reduction in APP detection on the cell surface as quantified in the graph. **d** Secreted soluble APP (sAPP) levels in the media from which each cell line had been incubated with for 24 h was also quantified by Western blotting using the same N-terminal APP antibody as in (**b**) to monitor total sAPP and an antibody that specifically recognizes amyloidogenically processed APP (sAPPβ; 1A9). Data are means ± SEM, n = 3 for experiments performed on three separate occasions, ***p < 0.001 compared to cells transfected with empty vector and ^###^p < 0.001 compared to APP^WT^ cells. All were analyzed by two-tailed t-tests

Colonies from single stably transfected N2a neuroblastomas were selected for empty vector control (Hyg), APP^WT^ and mutations lacking an extracellular phosphorylation (APP^S206A^) or N-glycosylation (APP^N467K^ and APP^N496K^) site (Fig. 1a). Colonies were selected based on APP expression in the cell, as determined by mRNA (data not shown) and total protein expression (Fig. 1b), only being increased by ~ 50% compared to endogenous APP in N2a (as shown by Hyg in Fig. 1b). Upon comparing the wild-type APP levels, it was shown that deletion of either the phosphorylation or N-glycosylation sites increased total cellular full-length APP levels (Fig. 1b).

APP is proposed to have its main functional role as a type 1 transmembrane protein on the cell surface. To interrogate the effect of these mutations on cellular location of APP, we further investigated cell surface levels (Fig. 1c), as well as the processing of APP through its downstream proteolytic pathways (Fig. 1d). Of the total APP in the cell, only a small fraction is known to localise to the cell surface. Despite only a small increase in total APP above endogenous levels in the APP^WT^ cell line, a ~ sixfold increase in the cell surface levels of APP^WT^ was measured by flow cytometry. Intriguingly, despite a retention of total levels of APP in the cell when the extracellular phosphorylation (APP^S206A^) or N-glycosylation (APP^N467K^ and APP^N496K^) site mutations were introduced into APP, the level of APP on the cell surface in all three cell lines was significantly reduced compared to APP^WT^ (Fig. 1c).

The lack of APP on the cell surface caused by the introduction of these PTM mutations could be through two mechanisms; either the premature form of APP is unable to be correctly transported to the cell surface and thus trapped inside the cell, or upon reaching the cell surface the ablation of these sites increases the processing of APP from the cell surface. To address this question, the level of the soluble APP (sAPP) was measured in the media (Fig. 1d). Both total sAPP and sAPP cleaved through the amyloidogenic pathway (to produce sAPPβ) were measured by Western. Compared to cells expressing only endogenous APP, overexpression of APP^WT^ increased sAPP production that paralleled the rise in total cellular APP levels (Fig. 1b). As sAPPβ levels were fourfold higher than in the endogenously expressing cell line, changes to total sAPP in this cell type were likely to predominantly come from amyloidogenic processing of APP. In contrast to the APP^WT^ cell line, deletion of the phosphorylation (APP^S206A^) or N-glycosylation (APP^N467K^ and APP^N496K^) sites caused a significant decrease in total sAPP compared to either the APP^WT^ or parental (Hyg) cell line. Despite this reduction of total levels of sAPP, when compared to the empty vector control only the N-glycosylation deletion mutants had reduced sAPPβ levels whereas APP^S206A^ was still partially increased. The secretion of APP being impaired by the deletion of these post translational sites infer that the lack of cell surface APP is likely to be caused by these mutations impairing APP trafficking to the cell surface.

### APP Mutants Alter Cellular Iron Processing Causing Retention of Iron

**Fig. 2:**
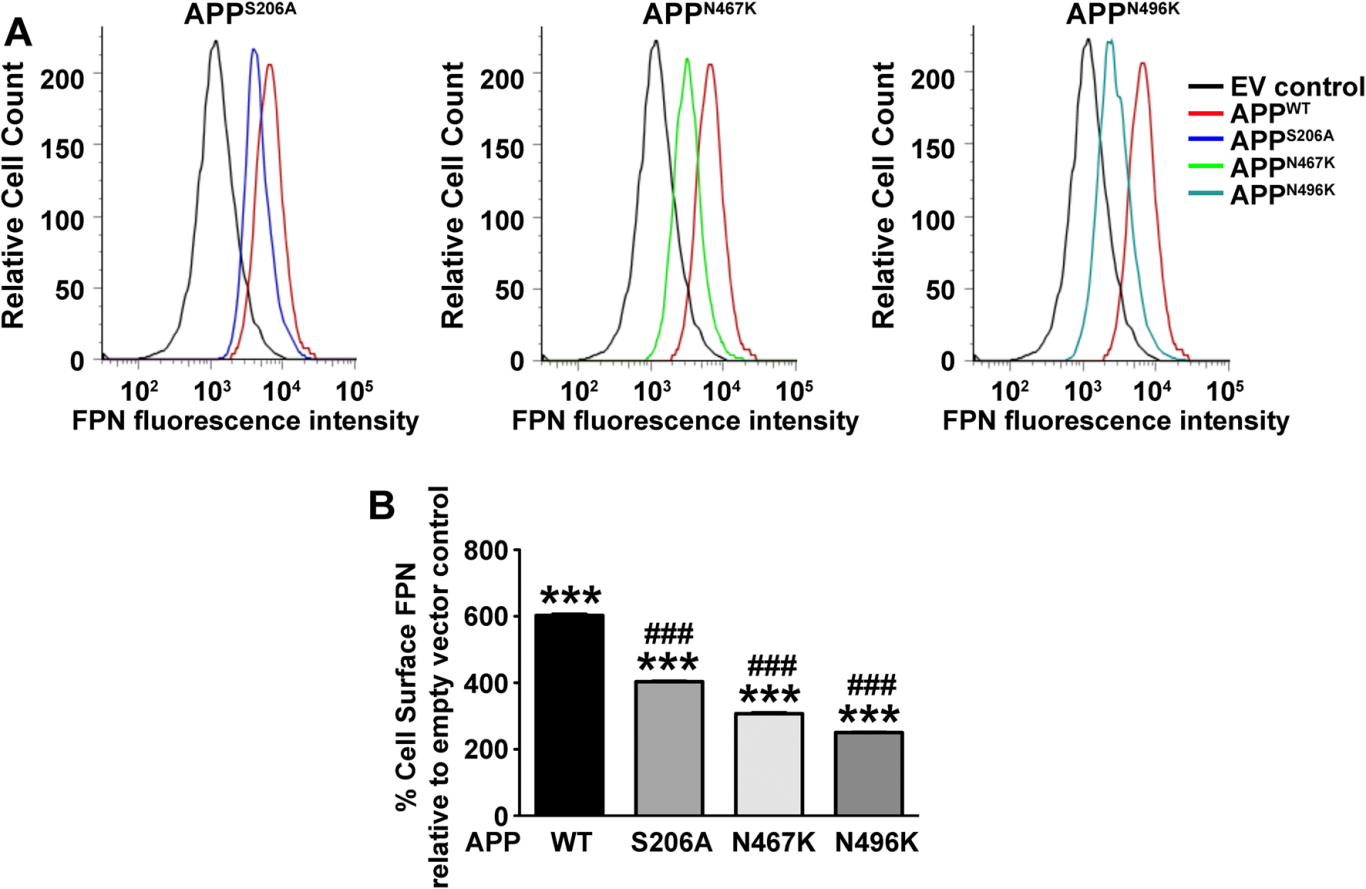
The effect of ectodomain N-glycosylation and phosphorylation site mutations in APP on stabilizing cell surface ferroportin. The role of APP in iron homeostasis is proposed to be through an ability to retain ferroportin (FPN) on the cell surface for iron efflux. **a** Representative histograms of flow cytometry measurements for cell surface FPN indicate a peak shift to the left in cell lines carrying APP mutations in a phosphorylation (S206A) or N-glycosylation (N467K and N496K) site compared to APP^WT^ overexpressing cell lines. **b** Quantification of the % change in FPN on the cell surface show that all APP overexpressing cell lines have elevated detectable FPN compared to the empty vector control line that only contains endogenous APP, but that FPN expression in cell lines with APP PTMs are significantly lower than APP^WT^. Data are means ± SEM, n = 3 for three separate experiments, ***p < 0.001 compared to cells transfected with empty vector and ^###^p < 0.001 compared to APP^WT^ cells. All were analyzed by two-tailed t tests

A proposed function of APP is to facilitate iron efflux by stabilising FPN on the cell surface [6–8]. Upon confirmation that deletion of extracellular phosphorylation (APP^S206A^) or N-glycosylation (APP^N467K^ and APP^N496K^) sites in APP modified its transport to the cell surface (Fig. 1), we investigated whether these changes had consequential effects on iron homeostasis. Comparable to cell surface APP levels upon overexpression of APP^WT^ (Fig. 1c), FPN was found to be substantially elevated in these cells (Fig. 2). The cell surface presence of FPN in cells expressing the phosphorylation or N-glycosylation mutations also correlated with surface APP levels, whereby these mutations reduced the FPN levels compared to APP^WT^, despite these still being higher than the empty vector control.

**Fig. 3:**
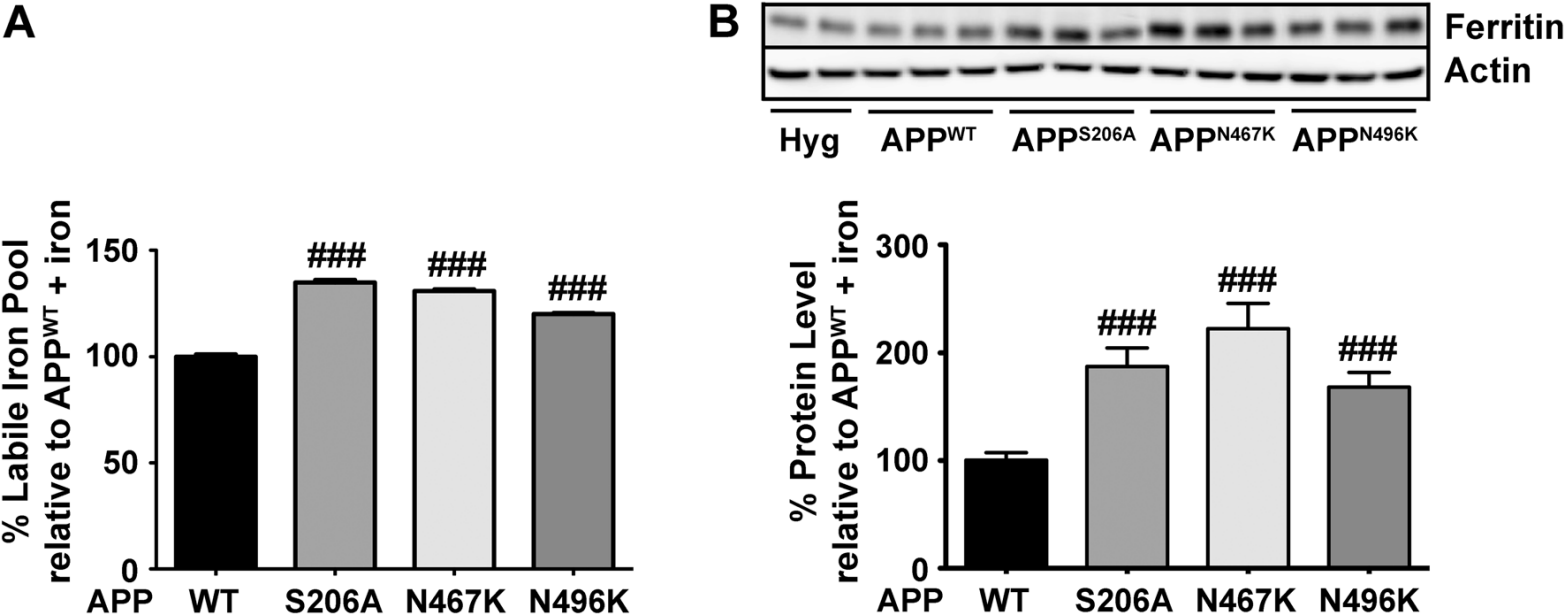
The effect of ectodomain N-glycosylation and phosphorylation site mutations in APP on intracellular iron homeostasis. **a** To directly measure the labile pool of intracellular iron a modified Calcein-AM assay quantified chelatable iron from cell lines containing post translational mutations compared to APP^WT^. All point mutations in APP caused a significant increase in iron within the cell. **b** Western blotting was used to quantify changes in expression of the iron responsive protein ferritin (FT) to indirectly confirm the altered iron homeostasis in each cell line observed in (**a**). Data are means ± SEM, n = 3 for three separate experiments, ###p < 0.001 compared to APP^WT^ cells. All were analyzed by two-tailed t tests

Cells were treated with iron (50 µM; 24 h) to determine if these mutations in APP altered the ability to regulate cellular iron levels due to an impaired capacity in stabilising FPN on the cell surface. Iron levels in the stable overexpressing cell lines after iron treatment were compared by directly measuring the chelatable labile iron pool (LIP) levels (Fig. 3a) and indirectly via expression of ferritin; an iron response protein (Fig. 3b). Both types of analysis confirmed that cells stably expressing the post translational mutants had an increase in cellular iron compared to cells overexpressing APP^WT^ (Fig. 3). Comparative iron relevant readouts to non-overexpressing cell lines were not analysed due to disparities between cells chronically expressing empty vector or APP^WT^ in a high iron environment. An elevated intracellular iron level in APP^WT^ cells compared to control (Hyg) (data in Fig. 3b not quantified) was considered to be a consequence of the APP^WT^ cell line having to adapt to survival under high iron efflux. However, this observation should not detract from the results that illustrate that the inhibition of a PTM to APP that is known to impair cell surface location of APP and FPN, can increase intracellular iron levels.

## Discussion

In a neuroblastoma model stably overexpressing APP, we provide support that PTMs are required for the trafficking of APP to the neuronal surface and further consolidate a proposed life cycle for APP. Mutations in the ectodomain of APP that ablate the correct N-glycosylation or phosphorylation of the protein lead to a retention of intracellular APP whilst also reducing the cell surface presence and subsequent secretion of APP. These observations illustrate a necessity in these PTMs to regulate the trafficking of APP to the cell surface and indicate how mature protein assembly by these modifications are a pre-requisite to proteolytic cleavage through either the non-amyloidogenic or amyloidogenic pathways. However, whilst there was a strong decrease to the surface location of APP in cells carrying these mutations it was also noted that levels of surface APP were still higher than the endogenously expressing parental line, indicating that trafficking had not been completely ablated but rather slowed. Similar observations have been previously observed [19] in which both impaired glycosylation and phosphorylation of APP were proven to result in the retardation of membrane trafficking rather than a reduction in surface APP half-life [25, 26]. N-glycosylation and ectodomain phosphorylation occur in discrete cellular compartments before it is trafficked to the cell surface. While we did not directly illustrate where APP accumulated within our cell model, prior evidence in non-neuronal cell lines strongly support mutations that impair N-glycosylation at these sites causing a retention of APP in the ER [15, 18–20] and mutations in ectodomain phosphorylation sites leading to accumulation in the TGN [21]. Of most relevance to this study, these prior reports agree that cell surface presence of APP is disrupted when these sites are mutated.

To date, the regulatory role of these PTMs on the function of full length APP has not been investigated. Instead, a greater focus has been on how these modifications effect downstream mechanisms such as the propensity to alter proteolytic processing and the production of cleavage products such as Aβ. Therefore, to our knowledge this is the first investigation into how these modifications regulate a known functional role of APP; the regulation of intracellular iron homeostasis. FPN is the only known iron export pore protein and must be localised on the cell surface for iron efflux to take place. APP is translationally regulated through an iron responsive element site in its promoter [27] and cell surface APP is increased in response to high intracellular iron [8]. Whilst FPN-dependent iron efflux can be modulated by several proteins (e.g. ceruloplasmin [28]), the absence of total APP in select cells including neurons has previously been shown to reduce levels of FPN on the cell surface [8] subsequently leading to intracellular iron retention [6, 7, 29]. However, it was unknown until now whether impaired maturation of APP would also have a consequential effect on cell surface FPN levels and regulation of intracellular iron. Outcomes from this study illustrate that disruption in the levels of APP on the cell surface caused by these post-translational mutations correlate with a reduction in surface FPN. This reduced capability to efflux cellular iron leads to an iron retention phenotype in these mutants compared to cells overexpressing wild-type APP. Despite total APP levels being increased in the APP mutant cell lines, the data underscores the importance of correct cellular trafficking for a functional role of APP on the cell surface. The iron retention caused by a permanent alteration in APP trafficking within these cell lines also highlight a possible mechanism by which the cell could modulate iron homeostasis through post-translational changes to APP.

The initial approach in the selection of which PTMs to study was based on whether these mutations would have a dual role in the protein; primarily in regulating the trafficking of APP to the cell surface and secondly if proximity of these sites to the FPN binding region in APP [6, 7, 29] were essential for stabilizing FPN on the cell surface. Outcomes have predominantly illustrated that these PTMs are essential for the correct location of APP for its functional role and therefore it is difficult to establish whether these sites are also required for FPN binding. However, it is worth noting that even if these PTMs were required for FPN binding their absence would be of little relevance if APP is primarily unable to be trafficked to the site of interaction on the cell surface.

This study cements an importance of APP trafficking in iron processing and indicates a regulatory nexus in cellular iron metabolism. Promising provisional outcomes provided here support further interrogation of the other PTMs known to occur with APP. This would provide greater clarity on the post-translational regulation of APP in iron efflux and whether interplay between the different types of modification can occur. Of relevance, APP is phosphorylated at several sites and whilst this study focused on only the ectodomain site at S206, this only accounts for 15% of APP ectodomain phosphorylation with S198 phosphorylation being a preceding factor [21] and heparin binding causing inhibition [22]. This points to a dynamic and transient interrelationship between all of the PTMs of APP to which this study has only just begun to scratch the surface.
